# Marked differences in the effects of levetiracetam and its analogue brivaracetam on microglial, astrocytic, and neuronal density in the rat model of kainic acid-induced temporal lobe epilepsy

**DOI:** 10.3389/fphar.2025.1553545

**Published:** 2025-03-06

**Authors:** Krisztina Kelemen, Máté Sárosi, Ágnes Csüdör, Károly Orbán-Kis, Hanga Kelemen, László Bába, Zsolt Gáll, Eszter Horváth, István Katona, Tibor Szilágyi

**Affiliations:** ^1^ Department of Physiology, George Emil Palade University of Medicine, Pharmacy, Science, and Technology of Targu Mures, Târgu Mureș, Romania; ^2^ Doctoral School, George Emil Palade University of Medicine, Pharmacy, Science, and Technology of Targu Mures, Târgu Mureș, Romania; ^3^ Molecular Neurobiology Research Group, HUN-REN Institute of Experimental Medicine, Budapest, Hungary; ^4^ Faculty of Medicine, George Emil Palade University of Medicine, Pharmacy, Science, and Technology of Targu Mures, Târgu Mureș, Romania; ^5^ Translational Behavioural Neuroscience Research Group, HUN-REN Institute of Experimental Medicine, Budapest, Hungary; ^6^ János Szentágothai Neurosciences Division, Doctoral College, Semmelweis University, Budapest, Hungary; ^7^ Department of Pharmacology and Clinical Pharmacy, George Emil Palade University of Medicine, Pharmacy, Science and Technology of Targu Mures, Târgu Mures, Romania; ^8^ Department of Psychological and Brain Sciences, Indiana University Bloomington, Bloomington, IN, United States

**Keywords:** temporal lobe epilepsy (TLE), antiepileptic drugs, microglia-activation, brain-regions, epileptic circuitry

## Abstract

Efficient treatment of temporal lobe epilepsy (TLE) remains challenging due to limited understanding of cellular and network changes and the interference of novel antiepileptic drugs (AEDs) with tissue reorganisation. This study compared the effects of brivaracetam and levetiracetam on histological alterations in key brain regions of the epileptic circuitry, namely, the hippocampus, amygdala, piriform cortex (PC), endopiriform nucleus (EPN) and paraventricular thalamic nucleus (PVT), using the kainic acid (KA) rat model of TLE. Male Wistar rats were assigned to sham-operated (SHAM), epileptic (EPI), brivaracetam- (BRV-EPI) and levetiracetam-treated (LEV-EPI) epileptic groups. Epileptic groups received KA in the right lateral ventricle, which induced status epilepticus followed by a 3-week recovery and latent period. Rats then underwent 3 weeks of oral brivaracetam, levetiracetam or placebo treatment with continuous video monitoring for seizure analysis. Subsequently, triple fluorescent immunolabeling assessed microglial, astrocytic, and neuronal changes. The results showed a drastic increase in microglia density in the EPI and BRV-EPI groups compared to control and LEV-EPI. The BRV-EPI group displayed a significantly higher microglia density than SHAM and EPI groups in the right CA1, CA3 and left CA1 regions, bilateral amygdalae, EPN, PVT and left PC. Astrocyte density was significantly elevated in hippocampal regions of the BRV-EPI group, while neuronal density decreased. Furthermore, brivaracetam did not reduce seizure activity in this disease phase. Significance: Brivaracetam treatment increased microglial activation under epileptic conditions *in vivo* in all examined brain-regions participating in the epileptic circuitry, in contrast to the effects of levetiracetam, highlighting differences in AED-induced histological alterations.

## 1 Introduction

Despite the numerous studies on its pathomechanism and treatment, epilepsy remains to date a disease of many faces that creates a significant global health burden (Beghi et al., 2019). Epilepsy substantially reduces the quality of life and uncontrolled seizures can pose a significant risk to the patient’s life. Mesial temporal lobe epilepsy (TLE) is among the most common forms of focal epilepsy, and approximately 30% of cases are resistant to antiepileptic drug (AED) treatment, creating a challenge for both clinicians and patients (Sultana et al., 2021).

The hippocampus has been well-studied in the context of TLE as the primary focus of the lesion. Moreover, several other brain regions have been shown to participate in the TLE epileptic circuit. For example, the amygdaloid regions are particularly sensitive to status epilepticus-induced neuronal damage, which often occurs alongside hippocampal injury (Ma et al., 2016). Furthermore, rodent and nonhuman primate studies provided evidence that structural and functional alterations, which appear due to seizure activity originating from the amygdala extend to other temporal lobe structures, such as the piriform cortex (PC) and the endopiriform nucleus (EPN) (Pitkänen et al., 1998). Among the subcortical regions, the thalamus–especially the midline thalamic nuclei-appear to be susceptible to TLE, possibly due to the extensive connections to the limbic system (Jo et al., 2019). Involvement in the epileptiform networks has also been suggested for the paraventricular thalamic nucleus (PVT), which bears substantial afferent connections with the amygdala (Kark et al., 2021). Although these brain regions, which have a significant vulnerability towards epilepsy, are particularly important for higher-order cognitive and emotional functioning (Pessoa, 2008) - and hence for the quality of life - the exact mechanisms through which TLE and different pharmacotherapeutic approaches affect them are still largely unknown. One hypothesis for how TLE can affect these brain areas is that many of the limbic sites have a direct monosynaptic connection from one to another, but there is also a potential indirect connection through the thalamus (Sloan et al., 2011a). This creates a divergent–convergent excitation amplification circuitry between these brain regions which in turn creates an excitatory drive in the target regions, enhanced by the cellular alterations caused by epileptic discharges (Sloan et al., 2011b; Bertram, 2014).

Epilepsy, depending on its type and etiology, can induce aberrant neuronal activity and network reorganization (Cavazos and Cross, 2006; Devinsky et al., 2018; Ofer et al., 2019). The kainic acid (KA) model is broadly considered as one of the most reliable and translationally relevant paradigms to study the cellular mechanisms underlying epileptic injury, because the cellular abnormalities are comparable to the histological pathology observed in human TLE (Rusina et al., 2021). For example, the pyramidal cells of the hippocampal CA3 region are the most vulnerable in the KA model. In addition, the piriform cortex is also susceptible to oxidative stress during status epilepticus (Vaughan and Jackson, 2014; Candelario-Jalil et al., 2001). Moreover, quantitative assessment of neuropathological changes in this model led to the identification of degeneration in the cerebral cortex, septal nuclei, olfactory bulb, hippocampus, thalamus, and hypothalamus, as well as the amygdala (Srivastava et al., 2020). Studies have shown that the distant alteration of these brain regions is not a consequence of the diffusion or intra-axonal transport of kainic acid but is causally related to the epileptiform activity (Ben-ari et al., 1980). Additionally, TLE is known to be associated with focal neuroinflammatory mechanisms leading to gliosis which are localised in very specific brain regions, possibly with a distinct vulnerability, and which can further contribute to the dysfunctional activity of the pathological network (Ortinski et al., 2010; Devinsky et al., 2013; Robel et al., 2015).

In addition to neuronal loss and aberrant neurogenesis, activated astrocytes and microglia undergo changes that can promote network hyperexcitability (Robel et al., 2015; Eyo et al., 2017; Victor and Tsirka, 2020). The KA model provokes similar local neuro-immune activation to that described in human epileptic brain tissue, highlighting the model’s feasibility for the global assessment of TLE-induced multidimensional brain injury (including neuronal damage and inflammatory response) and therapeutic intervention (Lévesque and Avoli, 2013; Wilson et al., 1996).

Despite the availability of over 20 antiepileptic drugs with various mechanisms of action, clinicians frequently encounter challenges in selecting the appropriate medication for their patients. These challenges are compounded by potential side effects, drug interactions, availability and individual response to treatment. The situation is further complicated by the incomplete understanding of these drugs’ mechanisms of action; most information pertains to their effects on neurons, while their specific impact on brain circuits remains largely elusive.

Levetiracetam (LEV) has become one of the most used medications in clinical practice due to its efficacy and low rate of adverse effects. Its clinical importance is highlighted by the fact that levetiracetam is listed by the World Health Organization as Essential Medicine. Levetiracetam functions by binding to the synaptic vesicle glycoprotein 2A (SV2A) (Crepeau and Treiman, 2010). In addition to its effectiveness in controlling seizures in both acute settings (e.g., status epilepticus) and chronic conditions, several studies have demonstrated that levetiracetam also exhibits suppressive effects on neuroinflammation and phagocytic microglia (Itoh et al., 2019). Brivaracetam (BRV), a novel antiepileptic drug approved by the FDA in 2016, shares a similar mechanism of action with levetiracetam but its affinity to SV2A is 15–30 folds higher (Klein et al., 2018; Gillard et al., 2011). SV2A is a glycoprotein and functions as a transmembrane protein and possibly as a transporter for galactoze (Bradberry and Chapman, 2024). Present ubiquitously in the CNS, SV2A is considered as a master regulator of vesicle trafficking and neurotransmitter release (Klein et al., 2018; Löscher et al., 2016). Loss of function of its gene is causing disturbance in GABAergic neurotransmission in the CA3 region (Crowder et al., 1999). The two drugs that target SV2A share the same binding pocket at molecular level but differ in terms of binding affinity both for SV2A and other molecular targets. This could explain (at least partially) the differences seen in the preclinical and clinical features of the two drugs. LEV is also a blocker of α-amino-3-hydroxy-5-methyl-4-isoxazolepropionic acid (AMPA) type receptors of glutamate and of high-voltage-gated calcium channels (N-type calcium channels) and an antagonist of 5HT3A receptors of serotonin while BRV is a blocker of voltage-dependent sodium channel in higher concentrations (Klein et al., 2018; Gillard et al., 2011). In supratherapeutic concentrations BRV is a weak inhibitor of NMDA-receptors, however the role of the latter mechanism is unclear (Matagne et al., 2008). It seems that BRV is a broad-spectrum AED, that has limited on-label use yet, and which has several advantages over other AEDs as it is recommended in numerous clinical situations by a recent Delphi consensus statement, such as early add-on for the elderly and for patients with post-stroke epilepsy or status epilepticus (Lattanzi et al., 2024). Furthermore, brivaracetam can be a safe option for patients with epilepsy who have psychiatric comorbidities and might not be good candidates for levetiracetam treatment (Zahnert et al., 2018). However, how brivaracetam treatment affects neuronal, astrocytic and microglial cellular organization remains unknown.

To address this issue, we systematically compared the effects of brivaracetam and levetiracetam on the microglia-astrocyte-neuron triad in TLE-associated brain circuits by using the rat KA model in the present study.

## 2 Materials and methods

### 2.1 Animals

All experiments were conducted in accordance with the 2010/63/EU Directive of the European Parliament as well as national regulations. The study was approved by the Ethics Committee for Scientific Research of the George Emil Palade University of Medicine, Pharmacy, Science, and Technology of Târgu Mureș (ethical committee license no. 43/06.03.2020, and no. 52/31.03. 2022).

The animals used in this study were experimentally naïve 8 weeks old male Wistar rats (∼200 g), which were provided by the Laboratory Animal Core Facility of the George Emil Palade University of Medicine, Pharmacy, Science, and Technology of Târgu Mureș (UMFSTGEP). Before the start of the experiments, all animals were subjected to a 5-day habituation period, when acclimatization to single housing, daily handling, in standard environmental conditions (12 h light-dark cycle, 20°C ± 2°C temperature, 60% ± 10% humidity) were carried out. The rats were housed in standard polypropylene cages (1291H Eurostandard Type III H, 425 mm × 266 mm × 185 mm, Techniplast, Milan, Italy). Standard rodent pellet chow (“Cantacuzino” National Institute of Research and Development, Bucharest, Romania) and tap water were provided *ad libitum*.

The aim of the study was to evaluate the effects of BRV and LEV in the KA model of temporal lobe epilepsy. Therefore, the animals were randomly divided into four experimental groups: kainic acid-injected–epileptic-control (EPI, n = 8), kainic acid-injected and brivaracetam-treated (BRV-EPI, n = 7), kainic acid-injected and levetiracetam-treated (LEV-EPI, n = 7); the rats from the sham-operated control group were administered saline solution instead of KA (SHAM, n = 7). As there is no available data for the brivaracetam’s effect on cell densities, a *post hoc* analysis based on the obtained r^2^s was performed with α = 0.05 and the used sample size (29 rats), which yielded a power (1 - β err prob) of 0.96, considered more than sufficient (G*Power v3.1.9.7, Universität Düsseldorf, Düsseldorf, Germany).

### 2.2 Epilepsy induction and video monitoring

The KA model of temporal lobe epilepsy was used, as it imitates the core aspects of human TLE with a standard sequence of events as follows–initial injury, latent period, chronic epilepsy with increasing seizure severity and frequency (Rusina et al., 2021). We performed intracerebroventricular (ICV) administration of KA, as it has lower mortality and less comorbidities when compared to systemic KA administration (Rusina et al., 2021).

KA was injected stereotaxically (Digital Stereotaxic with Manual Fine Drive, Leica Biosystems, Buffalo Grove, IL, USA; heating pad by Physiological Temperature Controller TMP5b, Supertech, Pécs, Hungary) with a 10 μL Hamilton syringe (Hamilton Company, Nevada, USA) under continuous anaesthesia (EZ-SA800 Single Animal Anesthesia System, World Precision Instruments Inc., Sarasota, USA) induced by 5% isoflurane (Anesteran, Rompharm Company SRL), with a 0.5%-2.0% maintenance dose, based on monitorization of anesthesia depth. The injection syringe was positioned according to The Rat Brain atlas of Paxinos and Watson (Paxinos et al., 2007), with bregma as a reference. The right lateral ventricle was targeted (AP: −0.7 mm, ML: +1.5 mm, DV: 3.6 mm to skull surface), the injection speed was set to 0.4 μL/min using a motorized programmable stereotaxic injector (Stoelting QSI Model 5311, Stoelting Co., Wood Dale, IL, USA). In the case of epileptic-control and AED-treated epileptic experimental groups, 0.6 μg of KA (Sigma Aldrich, St. Louis, MO, USA) dissolved in 2 μL saline solution was injected. In the case of the sham-operated group, 2 μL saline solution was injected instead of KA.

After injection, the needle was kept in position for 10 min to prevent reflux of the administered solution (Kraska et al., 2012; Knezovic et al., 2018). The KA-treated animals started to show tonic-clonic jerks immediately after recovery from anaesthesia, which resulted in status epilepticus (SE), defined as continuous seizures that lasted longer than 30 min. The animals that received only saline solution did not show seizure activity. Seizures were classified according to the revised Racine scale (rRS); based on video recordings Racine 1 stage seizures cannot be detected accurately, thereby we quantified Racine 2-6 stage seizures only (Lüttjohann et al., 2009; Cela et al., 2019). Only those animals, which had Racine 5 and/or Racine 6 grade seizures during SE were included in the study. SE was not stopped with medication and subsided after 2-3 h. The mortality rate was approximately 30% during SE in the acute phase. Postoperative anti-inflammatory treatment was avoided to prevent interference with a possible inflammatory glial response, in accordance with the study protocol approved by the ethical committee. After recovery the animals received i.p. rehydration, were individually placed standard Plexiglas cages, each with an individual motion detection HDCVI camera system and were continuously video monitored (24/24 h, 7 days/week) lasting throughout the whole study (DHI-XVR4104HS, 30 fps, resolution 1024x768, Dahua technology, Hangzhou, China). Video monitoring data was manually analyzed by experienced reviewers blind to the treatment group to detect spontaneous seizures and behavioural abnormalities. The registered seizures were classified according to the rRS, and grade 2 to 6 (R2-R6) seizures were quantified.

### 2.3 Drugs and Reagents, treatment protocol

After a 21-day post-surgical recovery and latent period, the animals from the treated epileptic experimental groups received either p.o. brivaracetam (60 mg/kgbw) or p.o. levetiracetam (70 mg/kgbw). All AED doses were selected based on previous studies. Lee et al. showed that a dose of LEV greater than 50 mg/kg is needed to protect against neuronal damage induced by status epilepticus (Lee et al., 2013). Similarly, BRV was demonstrated to reduce the development of posttraumatic epileptiform activity within a dose range of 21–100 mg/kg (Ling et al., 2022). Considering earlier results regarding the pharmacokinetics of the drugs in experimental animals, the drug-containing pellets were auto-administered twice daily (12-h time intervals) (Matagne et al., 2008; Dupuis et al., 2015; Klitgaard et al., 1998; Yan et al., 2005). In order to minimize the stress related to immobilisation and forced administration, the drugs were orally auto-administered by the animals, in drug-containing pellets, aided by an automated, in-house drug-pellet distribution system as previously described (Bába et al., 2020).

**FIGURE 1:**
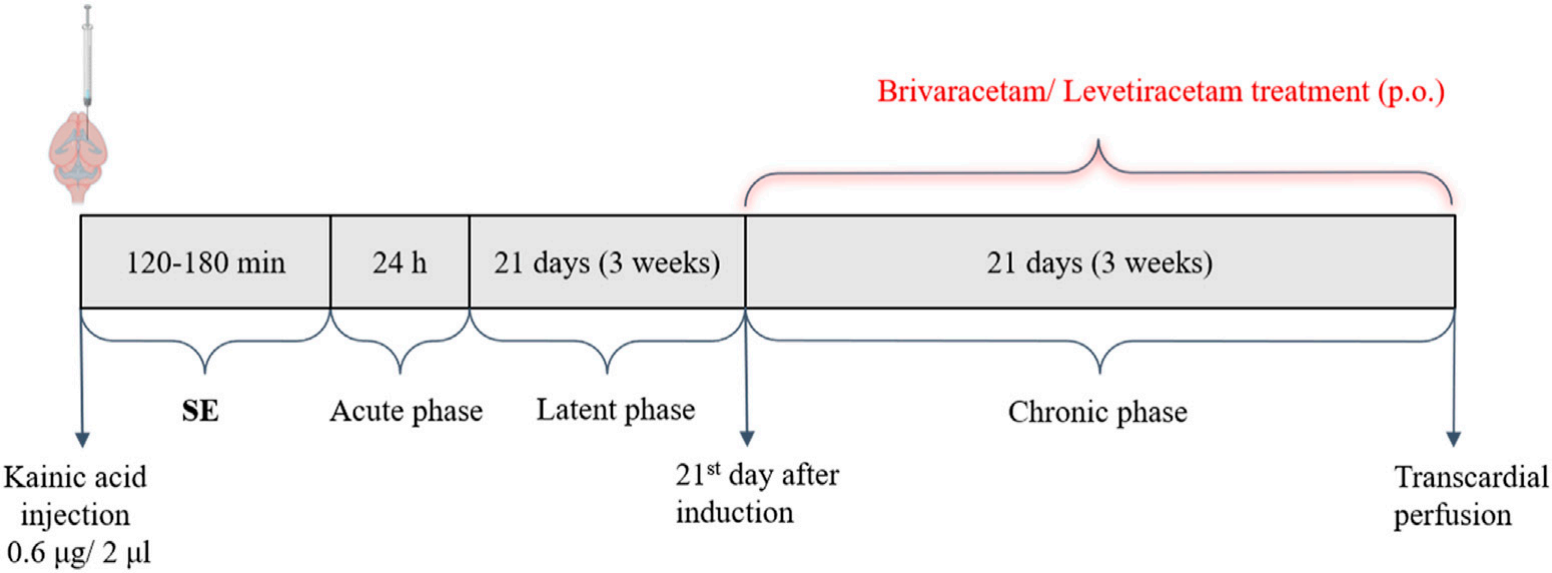
Timeline illustration of the experimental protocol. Brivaracetam/Levetiracetam were administered p.o. each day, starting on the 21st day after induction. Abbreviations: SE, status epilepticus.

The drug dose was calculated based on the animal’s body weight and dispersed in a mixture of lactose and glucose powder (1 g each). A few drops of glucose syrup were added to the mixture to obtain the required consistency and divided to obtain the drug-containing pellets (∼0.3 g each). These were loaded into the automated system and administered twice daily. The consumption of the drug-containing pellets was checked daily. None of the rats refused these pellets throughout the experiment. Furthermore, the plasma concentration of the antiepileptic drugs was determined in animals randomly selected from both treatment groups to ensure the dosing protocol’s consistency. Standard laboratory chow and tap water were available *ad libitum* throughout the experiment. The study period was preceded by a 5 days-long training period during which the animals were conditioned to the consumption of drug-containing pellets. After this accommodation period, the AED-treated epileptic animals received either BRV or LEV p.o. treatment for 3 weeks, as a 2-4-week treatment duration is generally regarded as useful to show both efficacy as well as the possible onset of tolerance to treatment (Leite and Cavalheiro, 1995; Glien et al., 2002; Löscher and Schmidt, 2006) This way the animal treatment model mimicked the human clinical setting. In the case of epileptic-control and sham-operated groups, the animals received vehicle-only pills. An overview of the experimental design is illustrated in Figure 1.

### 2.4 Immunohistochemical studies

#### 2.4.1 Fixation and brain sectioning

At the end of the 3 weeks long treatment protocol, a mixture of ketamine-xylazine (100 mg/kgbw and 10 mg/kgbw, respectively) was administered i.p. to the animals to induce deep anaesthesia. Rats from all experimental groups were perfused transcardially with ice-cold normal saline solution (0.9%, for 1.5 min), followed by ice-cold fixative solution containing 4% paraformaldehyde (Sigma Aldrich, St. Louis, MO, United States) and 15% picric acid (Sigma Aldrich, St. Louis, MO, United States) in 0.1 M phosphate buffer (PB, pH = 7.4, Sigma Aldrich, St. Louis, MO, United States) for 20 min. At the end of perfusion, the brains were removed from the skull and postfixed overnight in 4% paraformaldehyde (PFA). Subsequently, 60 μm-thick coronal sections were cut with a vibratome (VT 1000S, Leica, Nussloch, Germany) and washed in 0.1 M PB.

#### 2.4.2 Fluorescent immunolabeling

Triple fluorescent immunolabeling was used to simultaneously visualise microglia, astrocytes, and neurons, as well as the effects of seizures and the antiepileptic drugs on these cells. Free-floating brain sections containing the dorsal hippocampus were immunolabeled in a 24-well tissue culture plate (TPP, Trasadingen, Switzerland) in 500 μL volume on an orbital shaker (Heidolph Instruments, Schwabach, Germany).

After a 10-min wash in 0.1 M PB, and two times 10-min wash in Tris-buffered saline (TBS, Sigma-Aldrich, St. Louis, MO, United States), the sections were incubated in a TBS solution containing 10% normal horse serum (NHS; Vector Laboratories, Burlingame, CA, United States) and 0.3% Triton-X (Sigma-Aldrich, St. Louis, MO, United States) for 45 min, in order to block nonspecific binding sites as well as for membrane permeabilization and to enhance antibody penetration. Sections were subsequently incubated overnight with a primary antibody containing TBS supplemented with 0.1% Triton-X, at room temperature. The following antibodies were used: primary antibodies against NeuN for neurons (NeuN; guinea pig raised-polyclonal, dilution 1:500; product no: 266004, Synaptic Systems GmbH, Goettingen, Germany), GFAP for astrocytes (GFAP; mouse raised-monoclonal dilution 1:500; product no: 173211, Synaptic Systems GmbH, Goettingen, Germany), and IBA1 for microglia (IBA1; rabbit raised-polyclonal, dilution 1:500; HistoSure: HS234013, Synaptic Systems GmbH, Goettingen, Germany). Next day, the sections were washed thoroughly in TBS and the following fluorescently labelled secondary antibodies were applied at room temperature for 4 h: Alexa488-conjugated donkey anti-guinea pig (1:500, Jackson ImmunoResearch Laboratories, West Grove, PA, USA) to visualise the NeuN-immunolabeling, Alexa647-conjugated donkey anti-mouse (1:500, Jackson ImmunoResearch Laboratories, West Grove, PA, USA) to visualise the GFAP-immunolabeling, and Alexa594-conjugated donkey anti-rabbit (1:500, Jackson ImmunoResearch Laboratories, West Grove, PA, USA) to visualise the IBA1-immunolabeling. After incubation, the stained sections were washed in TBS and in 0.1 M PB, mounted on slides, coverslipped with a mounting medium (Vectashield, Vector Laboratories, Burlingame, CA, United States) and sealed with nail polish. All slides were stored at 4°C until imaging.

### 2.5 Confocal image acquisition and analysis of fluorescent immunolabeling

A Leica TCS SP8 confocal laser scanning microscope (Leica Microsystems GmbH, Wetzlar, Germany) was used to obtain high-resolution z-stack images of the fluorescent immunostainings from the dorsal hippocampus. All images were acquired using HC PL APO CS2 20X/0.75 dry objectives and unidirectional scanning at 200 Hz. The obtained images were processed using Leica Application Suite X software (Leica Microsystems GmbH, Wetzlar, Germany), and using built-in measurement functions of ImageJ (https://imagej.net/ij/) and Ilastik 1.4.0 software (www.ilastik.org, Berg et al., 2019).

The density of the three major cell types were quantified on high-magnification images of 1024 × 1024 pixels, and in individually selected regions of interest (ROIs - 300 μm × 500 μm). Z-stack deepness was defined as 5 μm, each image comprising of three subsequent z-stack layers, resulting in 10 μm-deep recordings. NeuN-immunolabeling was used to identify the different hippocampal regions and layers, as well as the examined anatomical structures. Vasculature and tissue scars were excluded from the analysis. During image analysis autofluorescence and non-specific signals were eliminated using background subtraction.

ImageJ software was used for manual cell-counting by an experimenter blind to treatment groups. Ilastik 1.4.0. software was used for supervised pixel-level classification and validation of previous results. Four slides per animal, and seven to eight animals per group were used for histological assessment. Microglia and astrocytes in the CA1 and CA3 regions of the hippocampus were invariably counted in the same area in all slices. The density of different cell types was expressed as cells/10^5^μm^2^ in the case of all examined regions. NeuN cell density in the CA1 and CA3 regions of the hippocampus was quantified on pyramidal layer lengths and expressed as cells/100 μm.

### 2.6 Slide scanner image acquisition and analysis

Immunolabeled whole brain images were scanned with a 3DHISTECH Pannoramic MIDI II slide scanner (3DHISTECH Ltd., Budapest, Hungary). Fluorescent images were obtained with a Zeiss Plan-Apochromat ×20 dry objective and Cy3.5, FITC-Q and SR_LF635 filters. The regions of interest were identified using the Paxinos and Watson Rat Brain Atlas. The paraventricular nucleus of the thalamus, the amygdaloid complex, the piriform cortex and the endopiriform nucleus were analysed by manual cell-counting. The three major cell types were quantified in all slices, and cell density was expressed as cells/10^5^ μm^2^.

### 2.7 Statistical analysis and figure preparation

Statistical analysis was performed with GraphPad Prism 8 software (version 8.0.1, GraphPad Software Inc., San Diego, CA, United States). All data are expressed as the mean ± standard error of the mean (S.E.M). Prior to statistical comparisons all data was checked using the ROUT (Robust regression and Outlier removal) method for multiple outlier detection. Kolmogorov–Smirnov test was used to test the data for normality and the appropriate statistical method. For matched observations, a two-way ANOVA followed by Tukey’s *post hoc* multiple comparison test was used. For unmatched groups and in case of data with a Gaussian distribution one-way ANOVA with Holm-Sidak’s multiple comparisons test was used. Otherwise, the Kruskal–Wallis nonparametric test with Dunn’s multiple comparisons test was used. An alpha value of 0.05 was used as the cutoff for significance. Sample size was checked for statistical power using G*Power (v3.1.9.7, Universität Düsseldorf, Düsseldorf, Germany).

Figure preparation was done with Gimp v2.10.32 (www.gimp.org), Adobe Photoshop CC 2015 and Microsoft PowerPoint. Confocal images of multiple samples and multiple experimental groups presented in the same figure were modified identically in every step to maintain the original differences.

## 3 Results

### 3.1 Brivaracetam differentially alters microglia, astrocyte, and neuron density compared to levetiracetam in multiple epileptic brain circuits

Because levetiracetam has a prominent effect on epilepsy and neuroinflammation-associated brain circuit reorganization, we first compared its effect with brivaracetam, a novel and more potent SV2A ligand, on glial and neuronal cell density changes by investigating brain regions that are especially vulnerable to kainic acid and could contribute to abnormal function of epileptic circuits.

**FIGURE 2:**
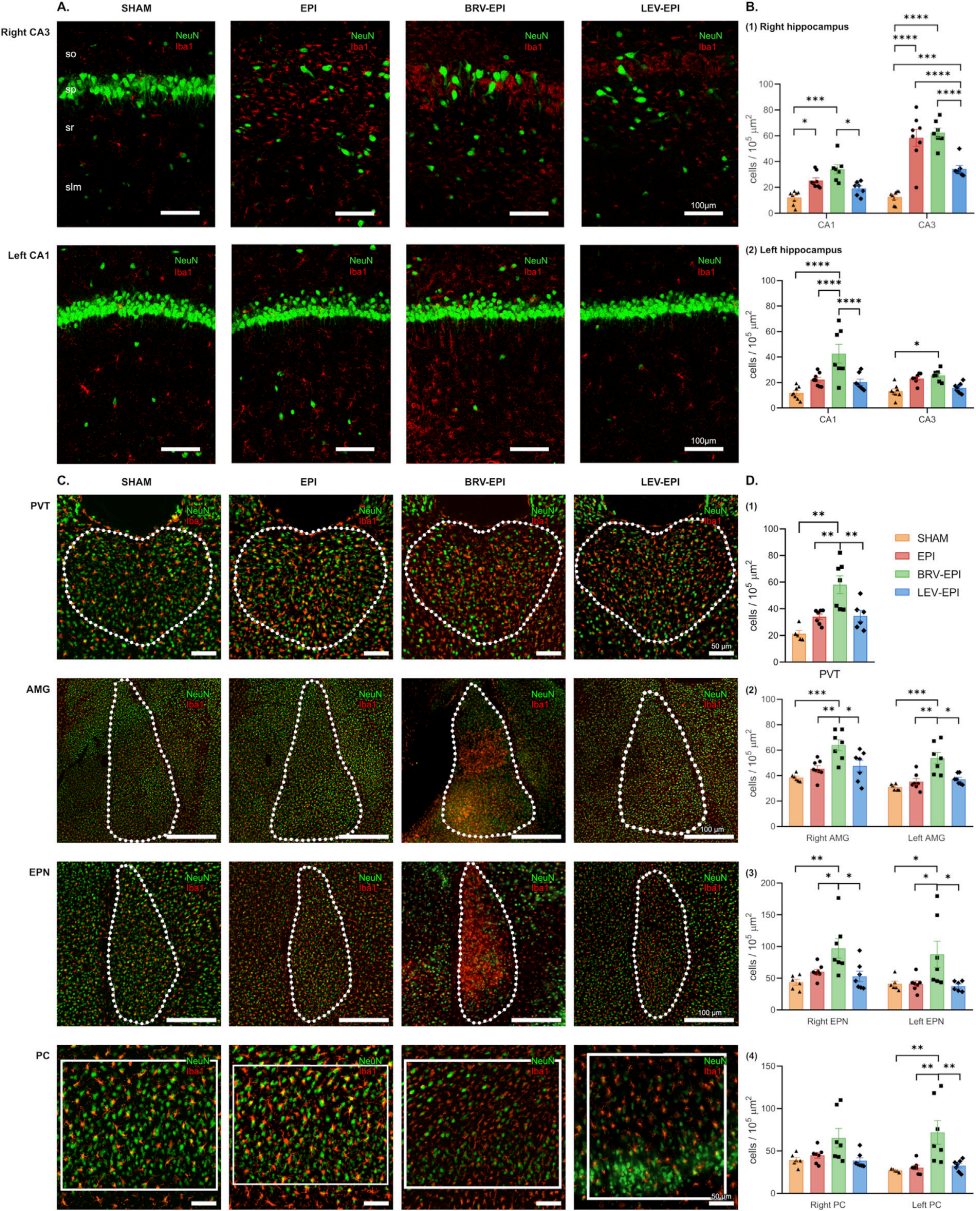
The effect of brivaracetam and levetiracetam on microglia cell density. **(A)** Fluorescent immunolabelling for neurons (NeuN) and microglia (Iba1) cells in the right CA3 (top row) and left CA1 (bottom row) areas; from left to right SHAM, EPI, BRV-EPI and LEV-EPI groups are presented. **(B)** Statistical analysis of microglia cell density in the right (B1) and left (B2) hippocampal CA1 and CA3 areas. *–p<0.05, ***–p<0.001, ****–p<0.0001. **(C)** Fluorescent immunolabelling for neurons (NeuN) and microglia (Iba1) cells in the PVT, AMG, EPN and PC brain regions. **(D)** Statistical analysis of microglia cell density in the PVT (D1), AMG (D2), EPN (D3) and PC (D4) brain regions. *–p<0.05, **–p<0.01, ***–p<0.001.

In the first set of experiments, microglia, astrocyte, and neuronal cell density was assessed in the hippocampal CA1 and CA3 regions as well as PVT, AMG, PC and EPN brain regions. Kainic acid exposure triggered a strong increase in microglia density in the right hippocampal CA3 region (which was located closer to the right ventricular site of the injection) 6 weeks following the initial insult (sham: 12.61 ± 1.71 cells/10^5^ μm^2^ versus KA: 58.46 ± 6.59 cells/10^5^ μm^2^ (Tukey’s *post hoc* test, p < 0.0001). Notably, levetiracetam treatment strongly attenuated this effect (34.38 ± 2.69 cells/10^5^ μm^2^ in the levetiracetam-treated group, Tukey’s *post hoc* test, p < 0.0001). In striking contrast, brivaracetam did not reduce the elevated microglia number (62.38 ± 3.67 cells/10^5^ μm^2^), when compared to the epileptic-control group (Tukey’s *post hoc* test, p = 0.9123). Moreover, there was a significant difference in microglial cell density between the brivaracetam and levetiracetam-treated groups (Tukey’s *post hoc* test, p < 0.0001, Figure 2A top row; Figure 2B1).

A similar effect was observed in the ipsilateral CA1 region (sham operated: 12.16 ± 1.83 cells/10^5^ μm^2^ vs. epileptic-control: 25.32 ± 2.12 cells/10^5^ μm^2^ vs. brivaracetam-treated: 34.11 ± 3.56 cells/10^5^ μm^2^ vs. levetiracetam-treated: 19.24 ± 1.97 cells/10^5^ μm^2^, Kruskal–Wallis test, p = 0.0002) (Figure 2B1).

While the contralateral CA3 was less affected by the KA injection and microglia density remained relatively unaffected within epileptic groups (sham operated: 13.17 ± 1.85 cells/10^5^ μm^2^ vs. epileptic-control: 23.23 ± 1.31 cells/10^5^ μm^2^ vs. brivaracetam-treated: 25.60 ± 1.71 cells/10^5^ μm^2^ vs. levetiracetam-treated: 15.91 ± 1.49 cells/10^5^ μm^2^, ANOVA, p = 0.19), a notable lesion was produced in the left CA1 region, contralateral to the site of the injection. Importantly, we found that KA injection triggered an increase in microglia density (F (3, 26) = 11.24, p < 0.0001) in the epileptic control group (22.21 ± 1.83 cells/10^5^ μm^2^) compared to the sham-operated (11.81 ± 1.74 cells/10^5^ μm^2^). Moreover, the effect of levetiracetam and brivaracetam treatment was also different in the contralateral side (20.29 ± 2.44 cells/10^5^ μm^2^ vs. 42.64 ± 7.36 cells/10^5^ μm^2^, respectively, Tukey’s comparison test p = 0.027, Figure 2A bottom row; Figure 2B2). In the brivaracetam-treated group an almost identical pattern of increased microglia density was seen in all the other examined brain regions, such as PVT (ANOVA, p = 0.0001), amygdala (right as well as left, ANOVA p < 0.0001), endopiriform nucleus (ANOVA, right p = 0.0027, left p = 0.0160) and piriform cortex (right side, ANOVA p = 0.0754; left side, Kruskal–Wallis p < 0.0025). Notably, in the case of the above-mentioned extra-hippocampal hemispheric structures, we could not detect lateralization in the extent of the microglia increase. Both hemispheres presented a substantial increase in microglia density in the brivaracetam-treated group compared to sham controls and even in solely kainic acid-treated rats. This unexpected finding suggests that brivaracetam treatment does not reduce epilepsy-associated microglia accumulation and the potential accompanying neuroinflammation (Figures 2C, D).

Next, we assessed neuronal cell density. KA injection into the right lateral ventricle strongly reduced the number of neurons in the right CA3 region (17.25 ± 0.72 cells/100 μm in the sham-operated group, 9.32 ± 1.6 cells/100 μm in the epileptic-control, 6.27 ± 0.79 cells/100 μm in the brivaracetam-treated, 8.54 ± 1.59 cells/100 μm in the levetiracetam-treated animals, Kruskal–Wallis test, p = 0.0064), accompanied by notable structural alterations of the pyramidal layer (Figure 2A top row; Figure 4A top row). The left CA3 region was relatively spared, the only significant difference in neuronal cell number was observed between the sham-operated group and the levetiracetam-treated group.

In contrast to the increase in microglia density we observed a substantial neuronal decrease in both CA1 regions in the brivaracetam treated group, when compared to the epileptic control (right side: 16.64 ± 1.81 cells/100 μm vs. 26.75 ± 2.37 cells/100 μm, p = 0.023; left side: 19.5 ± 3.2 cells/100 μm vs. 29.16 ± 1.78 cells/100 μm, p = 0.021).

**FIGURE 3:**
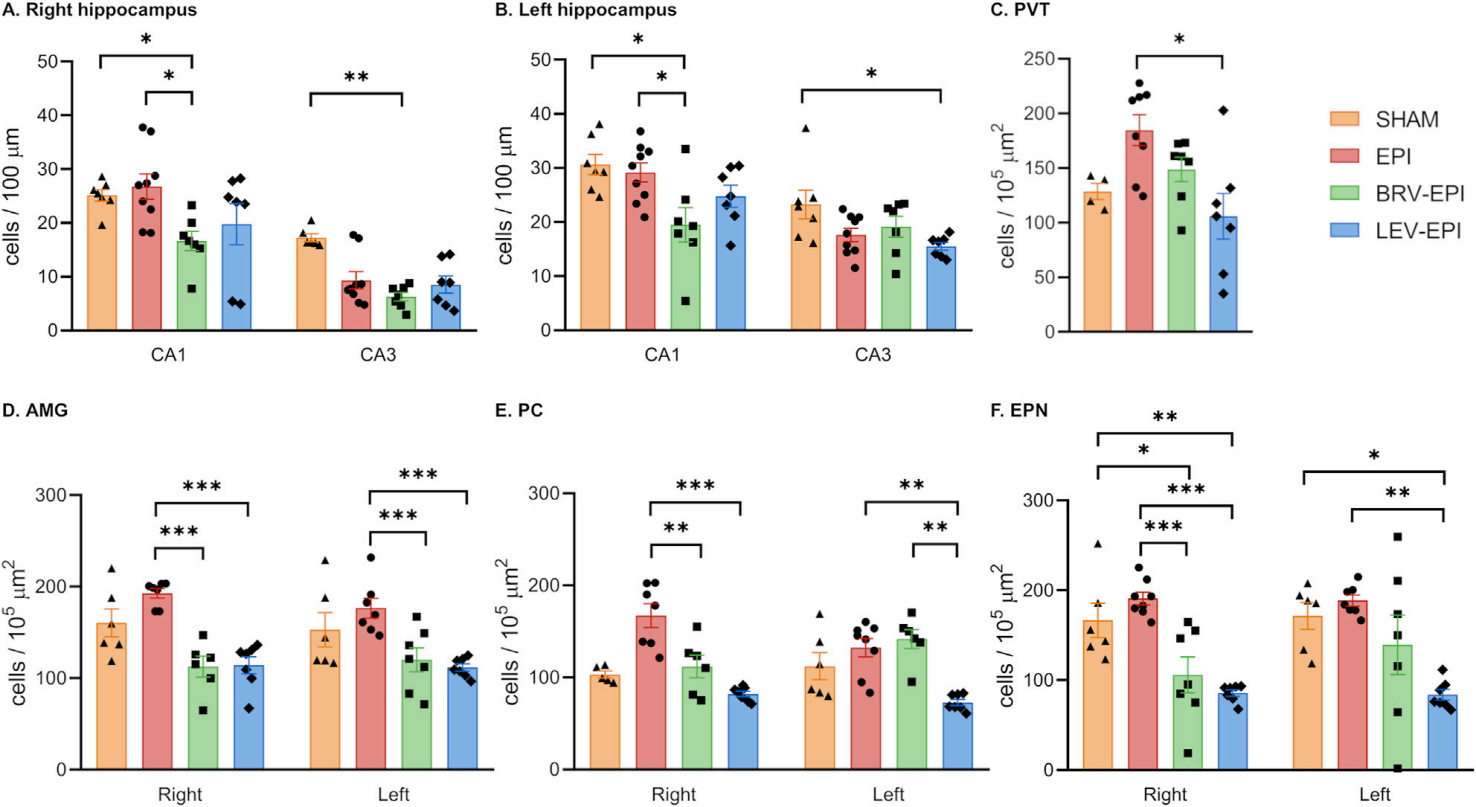
The effect of brivaracetam and levetiracetam on neuronal cell density. Statistical analysis of neuronal cell density in the right hippocampus **(A)**, left hippocampus **(B)**, paraventricular thalamic nucleus **(C)**, amygdala **(D)**, piriform cortex **(E)** and endopiriform nucleus **(F)** brain regions. *–p < 0.05, **–p < 0.01, ***–p < 0.001.

In addition, a significant decrease of neuronal density could be observed in both brivaracetam and levetiracetam-treated groups compared to the epileptic-control group in brain regions such as PVT (p = 0.0132), AMG (p < 0.0001 for both right and left side), PC (p < 0.0001 for both sides) and EPN (p = 0.0034 for left, p < 0.0001 for right side) (Figures 3A–F).

**FIGURE 4:**
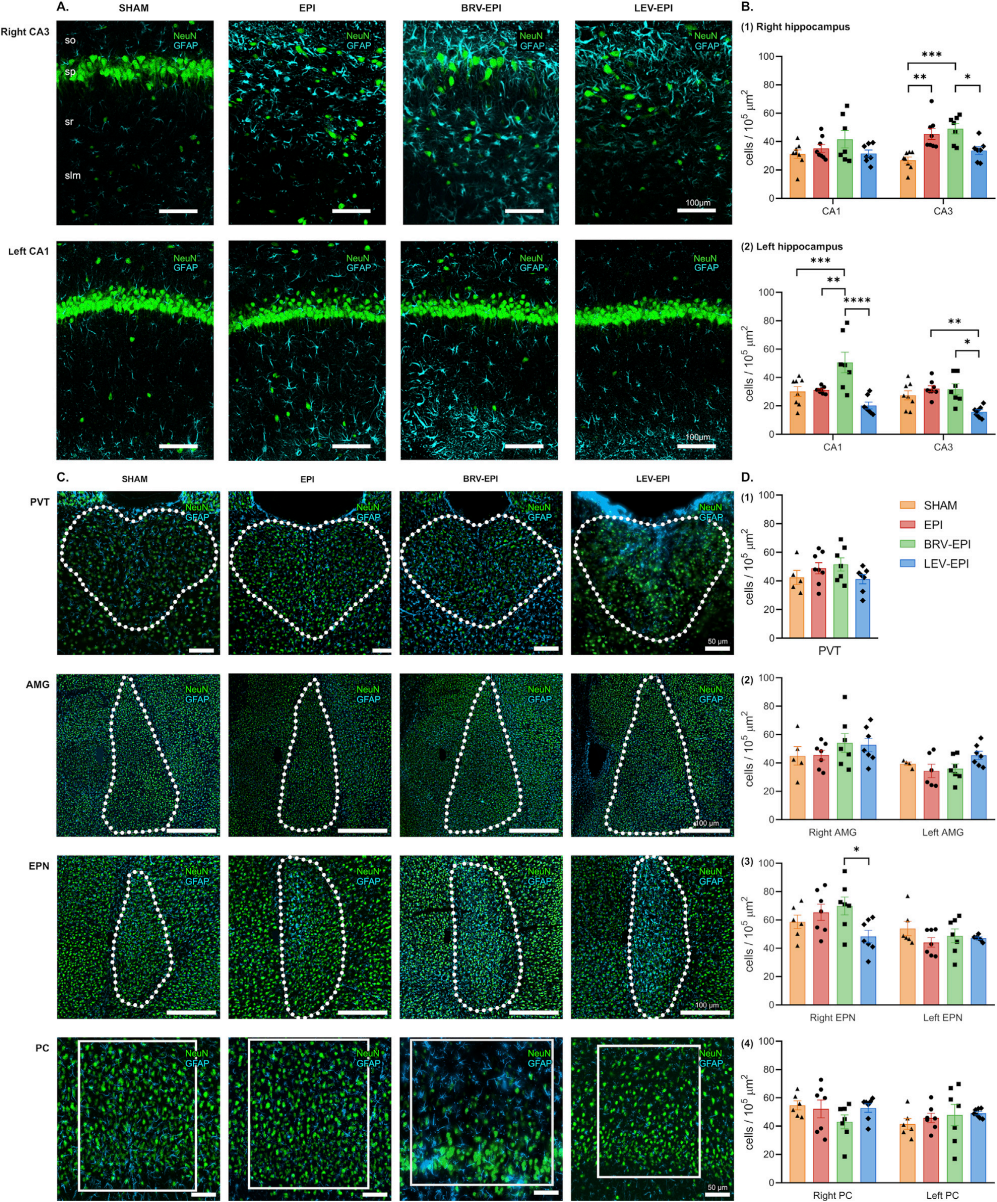
The effect of brivaracetam and levetiracetam on astrocyte density. **(A)** Fluorescent immunolabelling for neurons (NeuN) and astrocytes (GFAP) in the right CA3 (top row) and left CA1 (bottom row) areas; from left to right SHAM, EPI, BRV-EPI and LEV-EPI groups are presented. **(B)** Statistical analysis of astrocyte density in the right (B1) and left (B2) hippocampal CA1 and CA3 areas. *–p < 0.05, **–p < 0.01, ***–p < 0.001, ****–p < 0.0001. **(C)** Fluorescent immunolabelling for neurons (NeuN) and astrocytes (GFAP) in the PVT, AMG, EPN and PC brain regions. **(D)** Statistical analysis of astrocyte density in the PVT (D1), AMG (D2), EPN (D3) and PC (D4) brain regions.

Regarding astrogliosis, the hippocampus exhibited a prominent increase in the number of astrocytes, particularly in the right CA3 (26.91 ± 2.2 cells/10^5^ μm^2^ in the sham operated group, 45.37 ± 3.9 cells/10^5^ μm^2^ in the epileptic-control group, 49.05 ± 3.6 cells/10^5^ μm^2^ in the brivaracetam-treated and 33.71 ± 2.7 cells/10^5^ μm^2^ in the levetiracetam-treated group F (3, 26) = 10.38, p = 0.0001). The locations of the lesions corresponded closely to the regions exhibiting microglial activation, mainly the right CA3 (Figure 4A top row; Figure 4B1) and the left CA1 regions (Figure 4A bottom row; Figure 4B2) of the hippocampus (for left CA1: 30.21 ± 3.43 cells/10^5^ μm^2^ in the sham operated group, 31.23 ± 0.81 cells/10^5^ μm^2^ in the epileptic-control group, 50.64 ± 7.22 cells/10^5^ μm^2^ in the brivaracetam-treated, 20.29 ± 2.44 cells/10^5^ μm^2^ in the levetiracetam-treated group F (3, 26) = 9.39, p = 0.0002). However, chronic levetiracetam treatment maintained the astrocyte density on a level similar to the sham-operated control group. In contrast, the brivaracetam-treated group exhibited a significant increase in astrocyte cell density (Figure 4).

Regarding the other examined regions, statistically significant changes were observed only in the endopiriform nucleus on the right side (69.89 ± 6.29 cells/10^5^ μm^2^ in the brivaracetam-treated group vs. 48.35 ± 4.37 cells/10^5^ μm^2^ in the levetiracetam-treated group, p = 0.0396) (Figures 4C, D).

### 3.2 Comparative analysis of the KA-induced cellular vulnerability and the effects of drug treatment in different brain regions

In all epileptic groups, treated or untreated, we observed a significant increase in microglia density compared to the sham-operated group, across all brain regions that likely contribute to epileptiform activity (Figures 2C, D).

**FIGURE 5:**
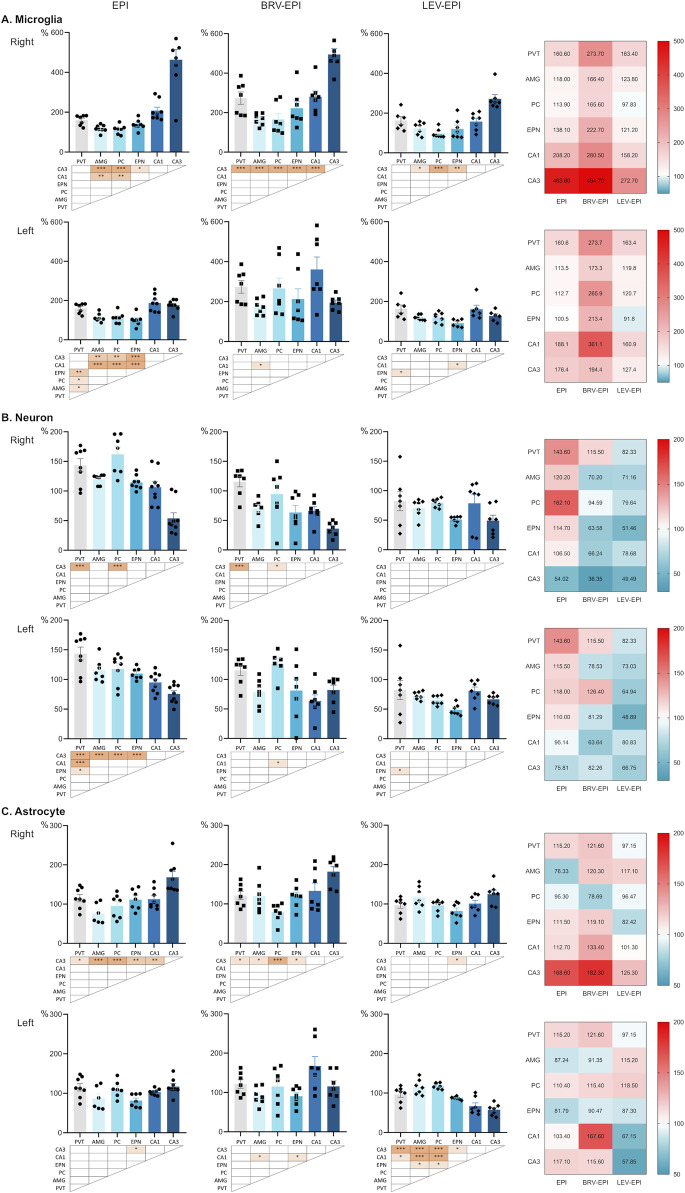
Distinct vulnerability of different brain regions in the epileptic circuitry. All panels show cell density across different brain regions normalized to the corresponding brain region of the sham operated group, expressed as percentage. Bar charts show cell densities separately for the right and left side for epileptic control (EPI), brivaracetam treated epileptic (BRV-EPI) and levetiracetam treated epileptic (LEV-EPI) groups. The notable exception is the midline nucleus PVT, which is repeated for both sides within an experimental group. Statistical comparison between brain regions is presented below each chart as *–p < 0.05, **–p < 0.01, ***–p < 0.001. Relative changes of cell densities are visualised as heatmaps on the right, where 100% (white) corresponds to the cell density of the sham operated group (for each side, brain region and treatment group). **(A)** Normalized microglia cell density. **(B)** Normalized neuronal cell density. **(C)** Normalized astrocyte cell density. Within an experimental group and cell type charts are scaled equally.

When we measured the changes in microglia density across different brain regions normalized to the corresponding brain region of the sham-operated group, the CA3 region in the right hippocampus exhibited the most significant increase in microglia density compared to other regions in all three epileptic groups (for a detailed statistical report see corresponding panels on Figure 5A top row). This region-specific increase might be attributed to the direct insult from the KA injection into the right lateral ventricle.

In the left hemisphere, the CA1 region of the hippocampus exhibited higher microglia density, in association with notable damage to this region. Additionally, the paraventricular thalamic nucleus demonstrated varying degrees of vulnerability across all experimental groups. In the brivaracetam-treated group, the piriform cortex and the endopiriform nucleus exhibited a more pronounced increase in microglial density. In contrast, in the levetiracetam-treated group, microglia density remained relatively consistent across the examined regions except from the primary lesion site (Figure 5A bottom row).

Regarding the modifications of neuronal cell density, the most pronounced neuronal loss also occurred in the CA3 region of the right hemisphere most likely due to KA injection into the right ventricle.

In the epileptic-control group, the neurons of PVT and the piriform cortex have been relatively spared compared to the hippocampus and the endopiriform nucleus. In the brivaracetam-treated group, aside from the hippocampus, the endopiriform nucleus and the amygdala seemed to have the most pronounced decrease of neurons (Figure 5B).

Conversely, in the levetiracetam-treated group, neuronal loss was more consistent across the examined regions, with fewer disparities observed. Nonetheless, the hippocampus and the endopiriform nucleus continued to exhibit the highest vulnerability, similarly to the other experimental groups, although overall changes in neuronal cell density were more consistent across regions (Figure 5B).

Upon comparison of the treated and untreated groups, notable changes in astrocytes were observed primarily in the hippocampus and the endopiriform nucleus, while other brain regions showed less consistent differences. Specifically, significant astrogliosis was observed in the hippocampus of the epileptic-control and brivaracetam-treated groups, whereas in the levetiracetam-treated group a pronounced astrogliosis was noted in the amygdala and piriform cortex (Figure 5C).

### 3.3 Effects of brivaracetam and levetiracetam on seizure frequency and semiology

**FIGURE 6:**
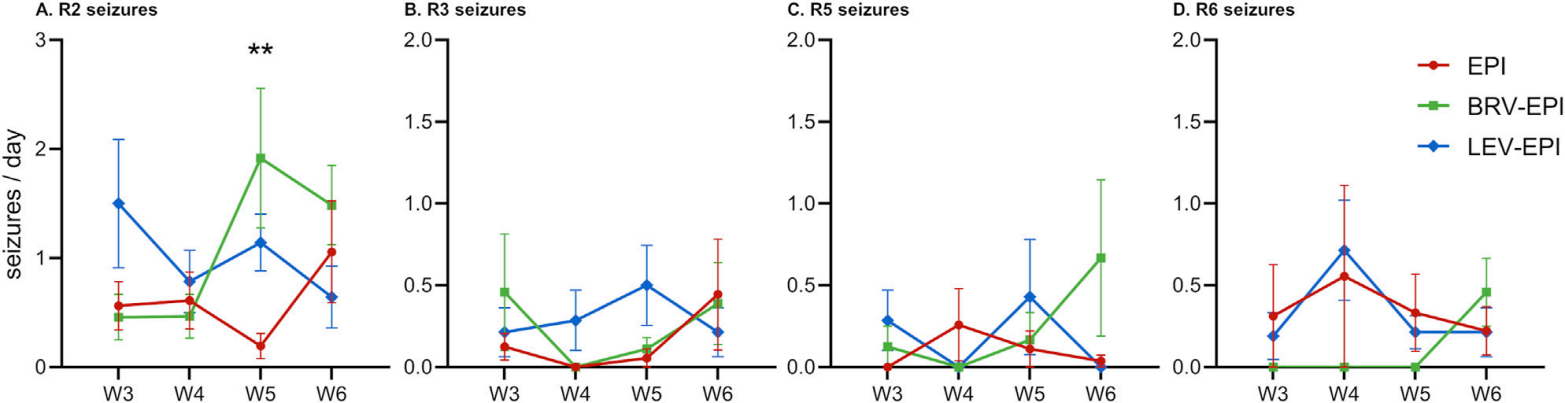
Effects of brivaracetam and levetiracetam on seizure density and severity. All panels show seizure density from the third to the sixth week of the study period (W3-W6) for epileptic-control (EPI), brivaracetam treated epileptic (BRV-EPI) and levetiracetam treated epileptic (LEV-EPI) groups. **(A)** Racine two severity seizures. **(B)** Racine three severity seizures. **(C)** Racine five severity seizures. **(D)** Racine six severity seizures. Note the different scale for **(A)** **–p = 0.003 for BRV-EPI vs. EPI.

Seizure frequency (seizures/day), severity (according to the rRS) as well as progression of seizures have been analyzed before and during the treatment period via 24-h video monitoring of the animals. It must be noted that all animals from EPI, BRV-EPI and LEV-EPI groups presented spontaneous recurrent seizures during the observation period. The frequency of R2 seizures was high in all epileptic groups (0.6 ± 0.18 seizure/day in the epileptic-control group, 1.08 ± 0.37 seizure/day in the brivaracetam-treated group and 1.02 ± 0.19 seizure/day in the levetiracetam-treated group) (Figure 6A). The frequency of mild seizures (R3) has slightly increased at 6 weeks in the epileptic-control and brivaracetam-treated groups (0.44 ± 0.34 seizures/day and 0.39 ± 0.25 seizures/day), whilst it has decreased in the levetiracetam-treated group (0.21 ± 0.15 seizures/day) (Figure 6B).

By the sixth week, the frequency of generalized seizures (R5) has dropped to zero in the levetiracetam-treated group, and slightly increased in the brivaracetam-treated group (0.67 ± 0.48 seizures/day), however this change was not statistically significant (BRV-EPI vs. EPI p = 0.0582, BRV-EPI vs. LEV-EPI p = 0.0573, see also Figure 6C). Furthermore, R6 seizures described as wild running and/or jumping have not been significantly affected by the antiepileptic treatment and seemed to be even increased in the brivaracetam-treated group (0.46 ± 0.21 seizures/day compared to 0.22 ± 0.15 in the epileptic-control group and 0.21 ± 0.15 in the levetiracetam-treated group) (Figure 6D). This pattern of seizures parallels the strong increase in the microglia density in the brivaracetam-treated group (Figures 2, 5, 6).

## 4 Discussion

Efficient treatment of temporal lobe epilepsy remains challenging, because of the incomplete understanding of the underlying cellular and network changes, especially in the hippocampus and other extra-hippocampal brain regions participating in pathological circuit activity. Antiepileptic drugs may induce glial and neuronal cellular changes *in vitro* (Ismail et al., 2022; Rogers et al., 2014), however, the mechanisms by which antiepileptic drugs interfere with the epilepsy-related region-specific, potentially maladaptive changes have remained largely unknown. In the context of temporal lobe epilepsy, the cellular alterations primarily include neuronal loss, astrocytosis, and neuroinflammation-associated microglia activation. However, the precise mechanisms by which antiepileptic drugs might modulate epileptogenesis and whether they can disrupt the vicious cycle associated with the underpinning cellular processes remains unclear.

This study aimed to quantitatively determine the impact of brivaracetam and levetiracetam treatment on neuronal damage and adaptive and maladaptive neuroinflammatory changes in the vulnerable brain regions implicated in TLE in the kainic acid model of temporal lobe epilepsy.

Intracerebroventricular injection of KA is a well-established rodent model for chronic temporal lobe epilepsy, effectively inducing spontaneous recurrent seizures and hippocampal sclerosis, corresponding to the clinical and morphopathological data derived from TLE patients (Ben-Ari, 1985; Rattka et al., 2013). Accordingly, KA injection induced status epilepticus in our experiments that later resulted in spontaneous seizures in all animals, making them suitable for studying the region-specific histological effects of TLE and the effects of AED treatment using brivaracetam and levetiracetam.

In our experimental model, we observed an increased microglial density 6 weeks after the initial insult in all analysed areas. This may be attributed to the persistent activity of the epileptic foci and, consequently, the continuous activation of microglia in these brain regions.

Microglial numbers are already increased in the hippocampus in the acute phase of intra-cerebral KA-induced seizures (the first 24-48 h post-induction) (Eyo et al., 2017). This prominent marker of inflammatory activation is accompanied by morphological alteration of microglia, characterized by the shortening and thickening of their processes and enlargement of their somata, conferring them a large, amoeboid shape. This has been associated with strong phagocytic activity and cytokine release (Eyo et al., 2017). It must be noted however that Iba1 is expressed in all microglial phenotypes (Amadio et al., 2014; Lier et al., 2021) and it does not differentiate between the various functional states of microglia, such as their phagocytic activity or inflammatory cytokine production. This suggests that while IBA1 is a great marker for quantitative and morphological analysis of microglial cells and its expression levels can provide valuable information, a combination of several markers will be necessary to precisely describe the functional state of these cells.

Furthermore, microglial activation has been reported to peak at 7 days following SE and to persist for at least 2 weeks (Yang et al., 2010). Although some studies have reported a transient decrease in microglia numbers around 2-3 weeks post-induction, most experiments have consistently shown a significant increase in the chronic phase, lasting for at least 5 months (Benson et al., 2015; Pernot et al., 2011; Upadhya et al., 2019). Microglial activation appears to be a secondary response to neuronal injury following the initial seizures and may be triggered not only by neuronal degeneration but also by neuronal hyperactivity preceding neuronal demise (Luo et al., 2016).

As microglia and other immune cells in the brain are known to contribute to neuroinflammation and phagocytosis during epileptogenesis, which is associated with spontaneous recurrent seizures (Luo et al., 2016; Itoh et al., 2016), it seems that microglial activation and increase in microglia numbers is linked to the progression of epileptogenesis (Victor and Tsirka, 2020).

In the epileptic brain, microglia appear to exhibit both pro-epileptic and anti-epileptic roles depending on the specific spatiotemporal context of the disease as well as on the stage of epileptogenesis (Lankhuijzen and Ridler, 2023; Kinoshita and Koyama, 2021). Despite their known pro-inflammatory roles, production of inflammatory mediators (such as cytokines*,* chemokines and complement proteins) that contribute to a cascade of inflammatory activity by activating downstream inflammatory genes (van Vliet et al., 2018; Vezzani and Granata, 2005) depletion of microglia in the mouse model of KA-induced epilepsy has been shown to increase seizure duration and intensity, elevate mortality, and exacerbate neurodegeneration in the temporal lobe (Wu et al., 2020).

Because microglia are highly plastic and can dynamically change their function and morphology in the context of their intrinsic and extrinsic immunological environment, they arise as important targets for antiepileptic treatment. AEDs can influence cell-mediated immunity; however, the underlying mechanisms are not well understood (Beghi and Shorvon, 2011). Since AEDs that were developed to target mainly neuronal mechanisms have not been efficient enough to reduce the proportion of patients with drug-resistant epilepsy, there has been an increasing focus on brain network components beyond neurons, with particular attention on microglia. Previous research has shown that various AEDs with different mechanisms of action have contrasting effects on microglia. For instance, valproic acid induced strong microglial activation under physiological and pathological conditions (Dambach et al., 2014).

In strong concordance with previous literature, we report in this study a significant decrease in microglia cell density in the levetiracetam-treated group compared to the other epileptic animals. Regarding levetiracetam, there seems to be a consensus. Prior studies have shown anti-inflammatory properties, as it inhibited the JNK/MAPK/NF-κB signaling pathway, which is associated with pro-inflammatory responses in microglia (Zhang et al., 2023), suppressed neuroinflammation and phagocytosis in a pilocarpine model of SE. (Itoh et al., 2019). Furthermore, levetiracetam decreased microglia activation and lowered pro-inflammatory cytokine expression (Victor and Tsirka, 2020; Itoh et al., 2016; Shima et al., 2018), possibly by targeting transcription factors like FosL1 (Niidome et al., 2021). It also reduced the pro-inflammatory interleukin-1β (IL-1β) level and promoted the expression of anti-inflammatory transforming growth factor-β1 (TGF-β1) under inflammatory conditions in a co-culture model (Haghikia et al., 2008; Stienen et al., 2011).

Although levetiracetam has been shown to have anti-inflammatory effects (Itoh et al., 2019), brivaracetam, a recently approved novel antiepileptic drug designed for high affinity and selectivity for synaptic vesicle protein 2A (SV2A) (Matagne et al., 2008), and practically a more potent analog of levetiracetam, has not yet been studied in the context of microglia activation *in vivo*. Intriguingly, treatment with BRV in therapeutic concentrations reduced the number of resting microglia, and increased microglial activation under inflammatory conditions, albeit in an *in vitro* astrocyte-microglia co-culture (Ismail et al., 2022). Even though it has been studied from multiple perspectives, brivaracetam’s effects in the KA model of epilepsy are not completely elucidated (Sanon et al., 2018).

In our experimental model, we have seen that in the brivaracetam-treated group, in regions vulnerable to KA injury, and in brain regions involved in the associated epileptic circuitry, there has been a marked increase in microglia cell density after 3 weeks of treatment. As previous studies that used double immunolabeling for SV2A and Iba1 have shown that microglia can also express SV2A (Tsymbalyuk et al., 2019; Makinde et al., 2020), our finding could be explained by the high affinity of BRV to SV2A, which in the case of higher medication doses, by binding to SV2A in microglia, could in turn induce the increase of microglia density, by influencing the proliferation of these cells (Rossi et al., 2022), or even as a secondary effect of knockdown apoptosis induced by SV2A (Yu et al., 2023). These findings are highly significant since they raise the possibility of a mechanism involving SV2A and microglia in an epileptic context, and even a potential pro-inflammatory effect of BRV.

In reference to changes in astrocyte cell density, astrogliosis was notably significant in the hippocampus for both the epileptic-control and brivaracetam-treated groups. Conversely, the levetiracetam-treated group showed pronounced astrogliosis in the amygdala and piriform cortex, indicating varying levels of astrocytic response across different brain regions.

Gliosis, characterized by the activation of microglia and astrocytes, is a histopathological hallmark of epilepsy (Devinsky et al., 2013), therefore the astrocytes are indispensable elements of epileptic tissue reorganizations and can contribute to the development of TLE (Henning et al., 2023). In the context of microglia-astrocyte interaction, the temporal sequence of their activation in epilepsy is controversial and may depend on the seizure model and induction protocol (Zhang et al., 2022; Maupu et al., 2021; Guo et al., 2022). In drug-induced SE models, an initial microglia activation has been shown to be followed by astrocyte activation (Sano et al., 2021), but SE itself can lead to astrocyte activation, which further activates microglia (Wei et al., 2021).

In the case of epileptic rats treated with brivaracetam/levetiracetam, there was a decrease in neuronal cell density. The relative increase of neuronal cell numbers in the case of epileptic-control rats, can be explained by the fact, that KA-induced status epilepticus has been demonstrated to generate a fivefold growth in NeuN-positive cells compared to control animals at 3 months post-SE (Jessberger et al., 2005). Moreover, the ectopically incorporated newborn neurons can participate in the process of epileptogenesis by forming recurrent excitatory networks in the dentate gyrus (Scharfman et al., 2000; Parent and Murphy, 2008; Koyama et al., 2012).

Although an initial significant neuronal loss likely occurred due to the neurotoxic effects of KA itself, the observed decrease in the number of NeuN-positive cells in the brivaracetam-treated group may be attributed to the enhanced microglial cell proliferation associated with this treatment. Activated microglia secrete tumor necrosis factor-alpha (TNF-α), which can inhibit the proliferation of neural progenitor cells in the SGZ following SE (Matsuda et al., 2015). Consequently, the increased microglial density observed after brivaracetam treatment may contribute to the reduction in NeuN-positive cells. Furthermore, activated microglia engulf post-SE-born cells via primary phagocytosis. Although microglia are essential in the restoration of injured neuronal tissue, after SE induction, or seizure, they can also damage living neurons by their phagocytic capacity or via cytokine release (Luo et al., 2016). On the other hand, other studies suggest that activated microglia are potentially neuroprotective against hyperexcitability-induced neuronal death (Araki et al., 2020), and by suppressing the uncontrolled proliferation of neural stem cells (NSCs) and eliminating adult-born granule cells, they restrain the generation of abnormal brain circuits after SE (Araki et al., 2021). The above hypothesis is supported by the fact that it was already demonstrated that levetiracetam limits neurogenesis in epileptic context and therefore also prevent the erroneous incorporation of newborn cells into the pathological circuitry (Sugaya et al., 2010). Moreover, in higher concentrations LEV has been shown to induce neuronal apoptosis (Zheng et al., 2022). Our results suggest that compared to the chronic epileptic group, where there could be an active neurogenesis and integration of new neurons occur, levetiracetam and brivaracetam have both exert a restraining effect on this process. This may indicate that different mechanisms with varying impacts may be responsible for decrease of NeuN + cells in these AED treated groups.

In contrast to the favourable behavioural outcomes previously reported for brivaracetam compared to levetiracetam (Sanon et al., 2018), our study found that brivaracetam, at the administered dose, did not reduce seizure activity in this phase of the disease. In fact, it appeared to increase the frequency of certain seizure types, parallel to the increase of microglia cell density. The seizure threshold can be reduced as a pathophysiological consequence of activated microglia, which in turn can result in epileptic seizures (Devinsky et al., 2013; Vezzani et al., 2011; Vezzani et al., 2013; Galic et al., 2012; Hiragi et al., 2018). This effect could be due to the fact, that BRV exerts dose- and age-dependent antiictogenic effects (Dupuis et al., 2015), moreover, resistance to treatment could also play a role, in the failure of relieving electroclinical seizures, as in the case reported by Jing Li et al., after 8 weeks of treatment. This latter effect was also described regarding other AEDs like lamotrigine, perampanel and carbamazepine (Li et al., 2023).

Limitations of this study include the use of only a single microglia-specific histological marker, IBA1, which did not allow the assessment of microglial phagocytic activity as well as the lack of investigation of the temporal sequence of glial and neuronal density changes. Also, a longer treatment period could allow the investigation of brivaracetams’ effects on microglia during chronic treatment. Additional studies are needed to investigate microglia activation in a dose-dependent manner using various drug doses, as well as to determine whether glial/neuronal changes can be prevented when BRV/LEV treatment is initiated immediately after SE. Furthermore, these results should be confirmed in different experimental *in vivo* paradigms.

## 5 Conclusion

We have shown that in a rat model of kainic acid-induced temporal lobe epilepsy, in the subacute phase, after 3 weeks of treatment with novel AEDs, there has been a significant difference between treatment groups in microglia cell density. Brivaracetam-treated animals showed an increased microglia density in anatomical structures contributing to epileptic activity (such as hippocampal CA3, CA1 regions, AMG, PC, EPN and PVT) compared to levetiracetam-treated animals. Furthermore, a prominent astrogliosis could be observed in the hippocampus particularly in the right CA3, ipsilateral to the KA injection, which was alleviated by levetiracetam compared to brivaracetam treatment. Also, a significant difference was observed in neuronal cell density between the epileptic and AED treated groups, with a relative decrease in NeuN cell numbers in the AED treated groups.

To effectively manage the elevated neuronal activity and inflammatory processes underlying seizures and subsequent epileptogenesis, it is crucial to deepen our understanding of microglia’s role, and that of glial cells in general, in epileptogenesis and AED treatment response. This insight could reveal mechanisms and targets for developing a new generation of antiepileptic drugs, and/or to associate anti-inflammatory medications in certain phases of the disease, focusing on microglia-mediated processes such as phagocytosis, aberrant neurogenesis, and neuroinflammation.

Moreover, for clinicians to achieve more targeted and personalized treatment, it may be reasonable in the future to fine-tune the selection of antiepileptic drugs based on the etiology of epilepsy, including those medications that can modulate microglial function.

## Data Availability

The raw data supporting the conclusions of this article will be made available by the authors, without undue reservation.
